# Inflammation and pro-resolution inflammation after hepatobiliary surgery

**DOI:** 10.1186/s12957-017-1220-6

**Published:** 2017-08-10

**Authors:** Juan P. Cata, Jose F. Velasquez, Maria F. Ramirez, Jean-Nicolas Vauthey, Vijaya Gottumukkala, Claudius Conrad, Bradford J. Kim, Thomas Aloia

**Affiliations:** 1 Department of Anesthesiology and Perioperative Medicine, The University of Texas MD Anderson Cancer Center, Houston, TX 77030 USA; 2 CES University, Antioquia, Colombia; 3 Department of Anesthesiology, Massachusetts General Hospital, Harvard University, Boston, MA USA; 4 Department of Surgical Oncology, The University of Texas MD Anderson Cancer Center, Houston, TX USA

**Keywords:** Inflammation, Resolution, Hepatobiliary surgery, Complications

## Abstract

**Background:**

The magnitude of the perioperative inflammatory response plays a role in surgical outcomes. However, few studies have explored the mechanisms of the resolution of inflammation in the context of surgery. Here, we described the temporal kinetics of interleukin-6, cortisol, lipoxin A4, and resolvin D in patients who underwent oncologic liver resections.

**Methods:**

All patients gave written informed consent. Demographic and perioperative surgical data were collected, along with blood samples, before surgery and on the mornings of postoperative days 1, 3, and 5. Interleukin-6, cortisol, lipoxin-A4, and resolvin D were measured in plasma. A *P* value < 0.05 was considered statistically significant.

**Results:**

Forty-one patients were included in the study. Liver resection for colorectal metastatic disease was the most commonly performed surgery. The plasma concentrations of interleukin-6 were *highest* on day 1 after surgery and remained higher than the baseline up to postoperative day 1. Postoperative complications occurred in 14 (24%) patients. Cortisol concentrations spiked on postoperative day 1. The concentrations of lipoxin A4 and resolvin D were lowest on day 1 after surgery.

**Conclusions:**

The inflammatory response associated with hepatobiliary surgery is associated with low circulating concentrations of lipoxin A4 and resolvin D that mirror, in an opposite manner, the kinetics of interleukin 6 and cortisol.

**Trial registration:**

NCT01438476

## Background

Increasing evidence indicates that inflammation has a significant impact on short- and long-term postoperative clinical outcomes. Several studies have found high circulating concentrations of interleukin-6 (IL-6) or C-reactive protein in patients who develop postoperative complications or die within 30 days of surgery [1, 2]. Kimura et al. demonstrated that patients who developed postoperative infectious complications after liver resection had significantly higher concentrations of IL-6 on days 0 and 1 after surgery than did those without infections [1].

Specialized pro-resolvin (Rv) mediators (SPMs) such as lipoxin A4 (LXA4), E-D series Rvs, protectins, and maresins are released in response to tissue injury and actively promote the resolution of inflammation [3]. Lipoxins and Rvs are formed in a process known as transcellular biosynthesis, which uses arachidonic acid and α-3 fatty acids as substrates [3]. Lipoxins and Rvs appear to be important mediators of the surgical stress response. In rodents, surgery-induced release of IL-6 was inhibited by aspirin-triggered RvD1 [4]. In an experimental model of peritonitis, the administration of fish oil (a natural source of eicosapentaenoic and docosahexaenoic acids) decreased the levels of IL-6 [5]. In patients who were undergoing major vascular surgery, two different patterns of inflammation and resolution were described on the basis of the temporal kinetics of SPMs: a pro-inflammatory pattern and a pro-Rv mediator profile [6].

To date, the temporal kinetics of SPMs in cancer patients who undergo major surgery remains unknown. Thus, in this study, we measured the plasma concentrations of IL-6, cortisol, LXA4, and RvD in patients who underwent oncologic hepatobiliary surgery. We hypothesized that low plasma concentrations of SPMs would be observed after surgery, particularly in patients who developed postoperative complications.

## Methods

### Ethics, consent, and permissions

After receiving institutional review board (IRB #2011-1046) approval and obtaining written informed consent from all patients, we collected demographic and perioperative data from an ongoing randomized controlled study of hepatobiliary surgery for primary biliary cancer or metastatic liver cancer (NCT01438476) at the University of Texas MD Anderson Cancer Center (Houston, Texas). We included all patients who underwent surgery between January 2013 and March 2015and were 18 years of age or older. We excluded patients who underwent emergency surgeries, those who were taking opioids chronically, pregnant women, and patients with major psychiatric conditions or immunologic disorders.

The anesthesia technique and postoperative pain management used in these patients was described elsewhere [7]. In brief, all patients underwent general balanced anesthesia, with or without postoperative epidural analgesia or intravenous patient-controlled analgesia. Surgical complication data were collected in real time and scored using the Accordion Severity Grading System [8]. Each complication was categorized into its appropriate system: neurology, cardiovascular, pulmonary, endocrine, metabolic, gastrointestinal, renal, hematology, wound, sepsis, and others. In addition, post-operative bile leak and liver failure were included for analysis using standard definitions [9, 10]. All complications and grades were verified by two staff members of the surgical oncology department at our institution.

Blood specimens were collected before surgery and on the mornings of postoperative days 1, 3, and 5. IL-6, cortisol, LXA4, and RvD concentrations in plasma were measured using an enzyme-linked immunosorbent assay and commercially available kits (R&D Systems, Inc., Minneapolis, CA, and MyBioSource, Inc., San Diego, CA). The assays were read in triplicate using an automated microplate reader (Molecular Devices, Inc., Sunnyvale, CA).

### Sample size calculation and statistical analysis

On the basis of previously published data [11] and using a type I error rate (α) of 5% and a power (1-β) of 80%, we estimated that 41 patients would be needed to demonstrate an increase of at least 50% in the plasma concentrations of IL-6 on day 1 after surgery.

Demographic, intraoperative, and postoperative data were analyzed and summarized using medians (interquartile ranges [IQRs]) or means (standard deviations or 95% confidence intervals) to account for outliers and non-normality in our description of study variables. The chi-square or Fisher’s exact test was used to compare categorical variables. The Friedman test, followed by Dunn’s multiple comparisons test, was used to compare preoperative and postoperative concentrations of IL-6, cortisol, LXA4, and RvD in the overall group of patients, and the Kruskal-Wallis test, followed by Dunn’s multiple comparisons test, was used to assess perioperative differences between patients with and without postoperative complications. A *P* value < 0.05 was considered statistically significant. The Spearman correlation test was used to assess the correlation between the concentrations of SMPs and IL-6. Prism 5 software (GraphPad Software, Inc., San Diego, CA) was used for all statistical analyses.

## Results

### Patients

Forty-one patients (22 male [54%]) with a median age of 57 years were included in the study (Table 1). Forty (98%) had an ASA physical status of 3. The median [IQR] heights and weights were 170 [163–176.5] cm and 78 [64.75–90.4] kg. Liver resection (*n* = 40 [98%]) for colorectal metastatic disease (*n* = 25 [61%]) was the most common surgical procedure. Fourteen patients (44%) developed postoperative complications; however, in 9, they were classified as moderate or severe (Accordion ≥ 2) (Table 2).

**Table 1:** Demographic and perioperative variables among 41 patients who underwent hepatobiliary surgery

Variable	Result
Age, years [IQR]	57 [48.5–67.5]
Sex, female/male, *n* (%)	22 (54)/19 (46)
Height, cm [IQR]	170 [163–176.5]
Weight, kg [IQR]	78 [64.75–90.4]
ASA physical status, *n* (%)	
1 or 2	1 (2)
3 or 4	40 (98)
Comorbidities, yes/no, *n* (%)	
Coronary artery disease	1 (2)/40 (98)
Hypertension	11 (27)/30 (73)
Hyperlipemia	4 (10)/37 (90)
Diabetes mellitus	3 (7)/38 (93)
Hypothyroidism	4 (10)/37 (90)
Medications, *n* (%)	
Statins	5 (12)/36 (88)
Antihypertensives	13 (31)/28 (69)
Antidiabetic drugs	3 (7)/38 (93)
Non-steroidal anti-inflammatory drugs	2 (5)/39 (95)
Histology, *n* (%)	
Cholangiocarcinoma	25 (61)
Gallbladder carcinoma	4 (10)
Colorectal carcinoma	2 (5)
Other	10 (24)
Preoperative chemotherapy, yes/no, *n* (%)	27 (66)/19 (34)
Type of surgery, *n* (%)	
Liver resection	40 (98)
Pancreatectomy	1 (2)
Duration of surgery (min), median [range]	293 [219.5–340.5]

Continuous variables are shown as medians [IQR, interquartiles]

*Cm* centimeters, *kg* kilograms, *ASA* American Society of Anesthesiologists

**Table 2:** Postoperative complications among 14 patients who underwent hepatobiliary surgery

Variable	Result
Estimated blood loss, mL [IQR]	250 [150–450]
Red blood cell transfusion, yes/no, *n* (%)	
Intraoperative	1 (2)/40 (98)
Postoperative	5 (12)/36 (88)
Complication type, yes/no, *n* (%)	
Neurologic	1 (2)/40 (98)
Cardiac	1 (2)/40 (98)
Respiratory	1 (2)/40 (98)
Renal	0 (0)/41 (100)
Gastrointestinal	4 (10)/37 (90)
Wound	2 (5)/39 (95)
Deep venous thrombosis/pulmonary embolism	2 (5)/39 (95)
Other	3 (7)/38 (93)
Highest Accordian grade, *n* (%)	
1	5 (36%)
2	5 (36%)
3	4 (28%)

*IQR* interquartiles

### Laboratory results

**Fig. 1:**
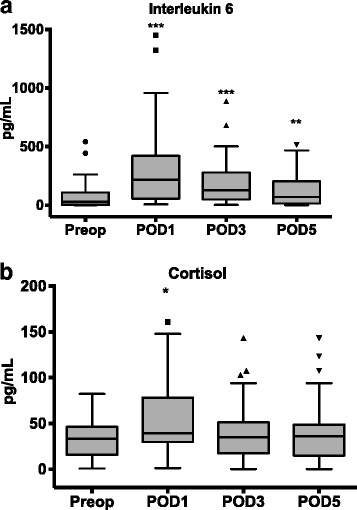
**a**, **b** Perioperative circulating concentrations of IL-6 and cortisol, respectively. Both markers showed a significant rise and peak on day 1 after surgery. *p < 0.05, **p < 0.01, and ***p < 0.001

As shown in Fig. 1a, the median (IQR) plasma concentrations of IL-6 were significantly higher on days 1 (216 [55.5–421] ng/mL, *p* < 0.001), 3 (128 [48.5–279] ng/mL), and 5 (70 [14–204] ng/mL, *p* < 0.001) after surgery than preoperatively (28 [2.5–109] ng/mL, *p* = 0.001). A Kruskal-Wallis test showed that the perioperative concentrations of IL-6 were slightly higher in patients with postoperative complications than in those without. However, no statistically significant differences were found for individual days (Table 3).

**Table 3:** Plasma concentrations of IL-6, cortisol, LXA4, and RvD in patients with and without postoperative complications

Cytokine	Median preoperative [IQR]	Median POD-1 [IQR]	Median POD-3 [IQR]	Median POD-5 [IQR]
IL-6, pg/mL				
Complications, *n* = 9	34 [19.33–46.25]	254 [82.25–593.3]	98 [54.75–268.3]	76 [50–284.8]
No complications, *n* = 31	27 [0–115]	206 [38–359]	128 [44–286]	59 [3–163]
*P* value	0.999	0.999	0.999	0.991
Cortisol, pg/mL				
Complications, *n* = 9	28.1 [13.60–40.66]	34.75 [31.19–87.6]	27.72 [8.46–46.2]	30.88 [16.4–48.3]
No complications, *n* = 29	33.34 [16.8–54.37]	40 [25.69–81.35]	39.12 [17.96–51.78]	40.36 [13.52–56.21]
*p* value	0.999	0.999	0.999	0.999
LXA4, pg/mL				
Complications, *n* = 9	3.95 [1.57–19.52]	3.2 [1.05–6.25]	3.15 [1.12–8.63]	2.87 [1.23–6.01]
No complications, *n* = 31	7 [2.01–17.24]	4.21 [1.48–8.99]	4.93 [1.68–10.64]	5.96 [2.27–13.63]
*p* value	0.999	0.968	0.999	0.534
RvD, pg/mL				
Complications, *n* = 9	1.13 [0.56–1.85]	0.43 [0.28–1.5]	0.76 [0.28–1.03]	1.02 [0.48–1.39]
No complications, *n* = 27	0.89 [0.6–1.5]	0.68 [0.47–1.03]	0.98 [0.67–1.93]	1.01 [0.69–2]
*p* value	0.999	0.435	0.501	0.999

*IL-6* interleukin 6, *LXA4* lipoxin A4, *RvD* resolvin D, *IQR* interquartile range, *POD* postoperative day

The concentrations of cortisol also increased after surgery (Fig. 1b), but only those measured on day 1 (39.32 [29.74–78.3] pg/mL, *p* = 0.04) were statistically significantly higher than those measured before surgery (preoperative: 33.17 [15.81–46.42] pg/mL vs. postoperative day 3: 34.74 [17.45–51.19] pg/dL, *p* = 0.999 and vs. postoperative day 5: 36.2 [14.78–48.83] pg/dL, *p* = 0.837). Patients with and without moderate or severe postoperative complications had similar perioperative plasma concentrations of cortisol (Table 3).

The circulating levels of LXA4 appear to mirror, in an opposite manner, those of IL-6. In brief, the median (IQR) plasma concentrations of LXA4 were significantly lower on postoperative days 1 (3.94 [1.36–7.45] ng/mL, *p* < 0.001), 3 (4.71 [1.61–9.39] ng/mL, *p* < 0.001), and 5 (5.02 [1.83–8.68] ng/mL, *p* = 0.006) than before surgery (7 [1.94–15.98] ng/mL). The Spearman correlation test demonstrated a significantly negative correlation (*r* = − 0.5 [95% confidence interval − 0.63 to 0.35], *p* < 0.0001) between the serum concentrations of IL-6 and LXA4. The postoperative concentrations of LXA4 were slightly but not statistically significantly higher in patients without complications than in those with moderate or severe (Accordion ≥ 2) complications (Table 3).

**Fig. 2:**
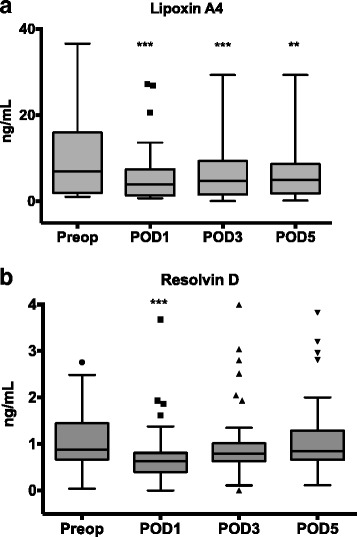
**a**, **b** Perioperative circulating concentrations of LXA4 and RvD, respectively. Both SPMs decreased on day 1 after surgery; however, the concentrations of LXA4 did not recover postoperatively. *p < 0.05, **p < 0.01, and ***p < 0.001

Similar to the kinetics followed by LXA4, we found that in comparison to preoperative values (0.88 [0.66–1.44] ng/mL), there was a significant decrease in the concentrations of RvD on day 1 (0.63 [0.39–0.81] ng/mL, *p* < 0.001) after surgery, but not on days 3 (0.79 [0.63–1.01] ng/mL, *p* = 0.298) and 5 (0.84 [0.66–1.29] ng/mL, *p* = 0.999) (Fig. 2b). In addition, a significant negative correlation (*r* = − 0.39 [95% confidence interval − 0.63 to 0.08], *p* = 0.11) was found between the serum concentrations of IL-6 and RvD. Although patients with moderate or severe (Accordion ≥ 2) complications had slightly lower RvD concentrations on postoperative days 1, 3, and 5 (Table 3), these differences were not statistically significant.

## Discussion

In our study, we found markers of inflammation and neuroendocrine stress response after surgery for primary biliary cancer or metastatic liver cancer, as evidenced by an increase in the plasma concentrations of IL-6 and cortisol.

Other investigators have demonstrated that IL-6 peaks after hepatobiliary surgery [12]. We found that IL-6 peaked on day 1 after surgery; however, there were no significant differences in the concentrations of cytokines between patients with and without complications. Conversely, Lahiri et al. demonstrated that the serum concentrations of IL-6 were statistically significantly different in patients with and without systemic inflammatory response syndrome after hepatobiliary surgery [12]. The discrepancies in the findings between Lahiri et al.’s work and ours are *presumably* due to differences in blood specimen collection days, outcome definitions, and unknown variables such as the anesthesia and analgesia technique used during and after surgery.

To the best of our best knowledge, this is the first study to investigate the temporal kinetics of LXA4 and RvD in oncologic patients undergoing surgery. In brief, we found that the plasma concentrations of LXA4 and RvD mirrored the perioperative kinetics of IL-6 and cortisol in a reverse direction. Specifically, the concentrations of LXA4 and RvD had reached their nadir on postoperative day 1, which coincided with the peaks in IL-6 and cortisol. The relevance of these findings remains unclear. It is possible that the initial postoperative decrease in plasma LXA4 and RvD, occurring at the same time as the peak in IL-6, is part of the stress response to surgical trauma (which is more intense than the lipopolysaccharide challenge), while the return to preoperative values represents the resolutive phase of surgery-induced trauma.

LXA4 and RvD are newly discovered molecules that function in the resolution of acute inflammation, pain, and sepsis. Both SPMs have important roles in a cascade of events that occur as part of the inflammatory and immune-suppressive response during and after surgery. For instance, LXA4 inhibits chemotaxis, neutrophil-epithelial cell interaction, natural killer cell function, and the radical of oxygen formation, while RvD is able to reduce the migration of neutrophils and the production of tumor necrosis factor [3]. Furthermore, there is increasing evidence that LXA4 has protective effects in animal models of systemic inflammation [13]. As an example, the administration of LXA4 in rodents with lipopolysaccharide-induced systemic inflammation significantly decreased the plasma concentration of IL-6 [13].

The roles that LXA4 and RvD play in recovery after surgery are largely unknown. Persistent low concentrations of SPMs after vascular surgery suggest a failure to resolve the inflammatory response and the presence of postoperative complications [6]. We were not able to show that patients who develop postoperative complications have higher plasma of levels of IL-6 and cortisol or lower circulating concentrations of SPMs than do those with an uneventful recovery. However, it is worth mentioning that our study was underpowered to show statistically significant differences in patients with and without complications. Also, we might have missed the nadirs or peaks of SPMs in our patients, with and without complications, because their timing depends on individual time courses of inflammation [14].

This study has several other limitations. First, we measured the levels of LXA4 and RvD using ELISA. It is well accepted that this is not the gold standard assay for determination of these molecules; however, a recent study by Fedirko et al. demonstrated a good correlation between the concentrations of LXA4 and RvD measured by spectrometry and ELISA (correlations of 0.88 and 0.83, respectively). Second, we only investigated the temporal kinetics of LXA4 and RvD. Therefore, the perioperative kinetics of other SPMs, such as RvE, protectins, and maresins, and their significance in postoperative recovery and relationship with morbidity, remain to be studied. Third, we included patients with a variety of cancers, comorbidities, and surgeries, which can influence the plasma concentrations of both proinflammatory cytokines and SPMs. We did not include a control group of subjects to compare their concentrations of LXA4 and RvD with those measured in our population of patients. Lastly, we did not consider the effects of drugs that can modulate the kinetics of SPMs, such as thiazolidinedione, aspirin, and fish oil, or the individual effects of different anesthetic techniques [15–17].

## Conclusions

The results of the present study demonstrate that the inflammatory response associated with hepatobiliary surgery is associated with low circulating concentrations of LXA4 and RvD. Understanding the profiles of the molecules that function in the resolution of inflammation can help identify patients who are likely to experience poor postoperative recovery. More studies are needed to elucidate the clinical relevance of these findings, particularly in the context of recovery after surgery.
